# Inhibition of Glutathione and Thioredoxin Metabolism Enhances Sensitivity to Perifosine in Head and Neck Cancer Cells

**DOI:** 10.1155/2009/519563

**Published:** 2009-09-02

**Authors:** Andrean L. Simons, Arlene D. Parsons, Katherine A. Foster, Kevin P. Orcutt, Melissa A. Fath, Douglas R. Spitz

**Affiliations:** Free Radical and Radiation Biology Program, Department of Radiation Oncology, Holden Comprehensive Cancer Center, The University of Iowa, Iowa City, IA 52242, USA

## Abstract

The hypothesis that the Akt inhibitor, perifosine (PER), combined with inhibitors of glutathione (GSH) and thioredoxin (Trx) metabolism will induce cytotoxicity via metabolic oxidative stress in human head and neck cancer (HNSCC) cells was tested. PER induced increases in glutathione disulfide (%GSSG) in FaDu, Cal-27, and SCC-25 HNSCCs as well as causing significant clonogenic cell killing in FaDu and Cal-27, which was suppressed by simultaneous treatment with N-acetylcysteine (NAC). An inhibitor of GSH synthesis, buthionine sulfoximine (BSO), sensitized Cal-27 and SCC-25 cells to PER-induced clonogenic killing as well as decreased total GSH and increased %GSSG. Additionally, inhibition of thioredoxin reductase activity (TrxRed) with auranofin (AUR) was able to induce PER sensitization in SCC-25 cells that were initially refractory to PER. These results support the conclusion that PER induces oxidative stress and clonogenic killing in HNSCC cells that is enhanced with inhibitors of GSH and Trx metabolism.

## 1. Introduction

Growing evidence exists that cancer cells are under increased intrinsic metabolic oxidative stress compared to normal untransformed cells due in part to mitochondrial dysfunction [1–3]. Studies have shown that defects in mitochondrial respiration led to activation of the Akt (protein kinase B) pathway, which may be an important mechanism by which cancer cells use to survive under conditions of chronic oxidative stress [4]. Akt is a serine-threonine protein kinase, which has been shown to have a role in angiogenesis, cell cycle progression, differentiation, and cell growth [5, 6]. Akt is hyperactivated in many cancer types including breast, colorectal, ovarian, and especially head and neck cancer (HNSCC) compared to normal tissue [7, 8], which led to the hypothesis that metabolic oxidative stress may be causally related to the increased Akt activity observed in cancer cells. Given that the Akt pathway is critical for cell survival, and cancer cells have been suggested to demonstrate increased intracellular hydroperoxide production compared to normal (untransformed) cells [2, 9–11], we propose that tumor cells may increase Akt activity to compensate for increased intracellular hydroperoxides and oxidative stress caused by defects in mitochondrial respiration. Furthermore, we propose that therapeutic interventions designed to inhibit Akt activation and hydroperoxide detoxification combined with manipulations that increase prooxidant production would preferentially kill tumor cells versus normal cells via oxidative stress. 

Perifosine (Octadecyl-(1,1-dimethyl-piperidinio-4-yl)-phosphate (PER)) is a bioavailable alkylphospholipid which is currently being tested in phases 1 and 2 clinical trials [12–17] and is a member of a larger group of membrane permeable single-chain antitumor alkylphosphocholines (APCs) [18–20]. PER has shown significant antiproliferative activity in in vitro and in vivo tumor models including breast, colon, prostate and HNSCC [18, 21]. Akt is a specific target of PER by targeting the plekstrin homology (PH) domain of Akt and preventing its translocation to the plasma membrane to be activated [21]. The aim of the present study is to determine if PER induces oxidative stress and if disrupting thiol antioxidant metabolism pathways further enhances sensitivity to PER-induced clonogenic cell killing in HNSCC cells.

## 2. Materials and Methods

### 2.1. Cells and Culture Conditions

FaDu, Cal-27, and SCC-25 human head and neck squamous carcinoma cells were obtained from the American Type Culture Collection (Manassas, VA). FaDu and Cal-27 cells were maintained in Dulbecco's modified Eagle's medium (DMEM) containing 4 mM L-glutamine, 1 mM sodium pyruvate, 1.5 g/L sodium bicarbonate, and 4.5 g/L glucose with 10% fetal bovine serum (FBS; Hyclone, Logan, UT). SCC-25 cells were maintained in a 1 : 1 mixture of Dulbecco's modified Eagle's medium and Ham's F12 medium containing 1.2 g/L sodium bicarbonate, 2.5 mM L-glutamine, 15 mM HEPES, 0.5 mM sodium pyruvate, 4.5 g/L glucose, and 400 ng/mL hydrocortisone with 10% fetal bovine serum. Cultures were maintained in 5% CO_2_ and humidified in a 37°C incubator.

### 2.2. Drug Treatment

N-acetyl cysteine (NAC) and L-buthionine-[S,R]-sulfoximine (BSO) were obtained from Sigma Chemical Co. (St. Louis, MO). Auranofin (AUR) was obtained from ICN Biochemicals (Aurora, OH). Perifosine (PER) was obtained from Cayman Chemical (Ann Arbor, MI). All drugs were used without further purification. Drugs were added to cells at final concentrations of 1–10 *μ*M PER, 0.5 *μ*M AUR, 20 mM NAC, and 1.0 mM BSO. PER and AUR were dissolved in ethanol and dimethyl sulfoxide (DMSO), respectively, and then diluted with 0.9% sodium chloride (Hospira, Lake Forest, IL), NAC was dissolved in 1 M sodium bicarbonate (pH 7.0) and BSO was dissolved in PBS. The required volume of each drug was added directly to complete cell culture media on cells to achieve the desired final concentrations. All cells were placed in a 37°C incubator and harvested at the time points indicated.

### 2.3. Detection of Activated Akt and Total Akt Levels

Cells were harvested after 24 hours drug exposure, washed with PBS, and then lysed with cell lysis buffer. Cell lysates were standardized for protein content and resolved by SDS-PAGE. Nitrocellulose blots were probed with rabbit antiAkt, antiphospho-Akt Ser^473^ (Cell Signaling Technologies, Beverly, MA), or antiGAPDH (Cell Signaling) antibodies.

### 2.4. Glutathione Assay

Cell pellets were thawed and homogenized in 50 mM PO_4_ buffer (pH 7.8) containing 1.34 mM diethylenetriaminepentaacetic acid (DETAPAC) buffer. Total glutathione content was determined by the method of Anderson [22]. Reduced glutathione (GSH) and glutathione disulfide (GSSG) were distinguished by addition of 2 *μ*L of a 1 : 1 mixture of 2-vinylpyridine and ethanol per 30 *μ*L of sample followed by incubation for 1 hour and assayed as described previously [23]. All glutathione determinations were normalized to the protein content of whole homogenates using the method of Lowry et al. [24].

### 2.5. Clonogenic Cell Survival Experiments

Cells were treated with PER for 24 hours prior to each clonogenic survival experiment. In the indicated experiments, cells were treated with NAC, BSO, or AUR for 1 hour before and during PER exposure. Attached cells from experimental dishes were trypsinized with 1 mL trypsin-EDTA (CellGro, Herndon, VA) and inactivated with DMEM containing 10% fetal bovine serum (Hyclone). The cells were diluted and counted using a Coulter counter. Cells were plated at low density (150–1000 per plate), and clones were allowed to grow in a humidified 5% CO_2_, 37°C environment for 14 days in complete medium, in the presence of 0.1% gentamicin. Cells were fixed with 70% ethanol and stained with Coomassie blue for analysis of clonogenic cell survival as previously described [25].

Individual assays were performed with multiple dilutions with at least four cloning dishes per data point, repeated in at least 3 separate experiments.

### 2.6. Thioredoxin Reductase Assay

Thioredoxin reductase (TrxRed) activity was determined spectrophotometrically using the method of Holmgren and Bjornstedt [26]. Enzymatic activity was determined by subtracting the time-dependent increase in absorbance at 412 nm in the presence of the TR activity inhibitor, aurothioglucose from total activity. One unit of activity was defined as 1 *μ*M TNB formed/(min·mg protein). Protein concentrations were determined by the Lowry assay [24].

### 2.7. Statistical Analysis

Statistical analysis was done using GraphPad Prism version 4 for Windows (GraphPad Software, San Diego, CA). To determine differences between 3 or more means, one-way ANOVA with Tukey posttests were performed. Error bars represent the standard error of the mean. All statistical analysis was performed at the *P* < .05 level of significance.

## 3. Results

### 3.1. Effect of Perifosine on Akt Expression

**Figure 1:**
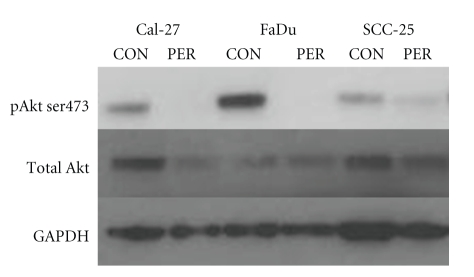
Effect of perifosine (PER) on Akt expression. Akt phosphorylation in FaDu, Cal-27, and SCC-25 cells was assayed by Western blots (50 *μ*g protein loaded in each well) for Akt and phosphor-Akt Ser473 in the presence or absence of 5 *μ*M PER for 24 hours. GAPDH was used as a loading control.

To determine the effect of PER on Akt expression in HNSCC, FaDu, Cal-27, and SCC-25 cells were treated with 5 *μ*M PER for 24 hours then harvested for the detection of activated Akt (pAkt) and total Akt. Activated Akt was detected in all 3 cell lines but varied in their expression levels (Figure 1). PER completely inhibited the expression of pAkt in FaDu and Cal-27 cells and partially inhibited pAkt expression in SCC-25 cells (Figure 1). Total Akt was also inhibited by PER in Cal-27 cells (Figure 1). These results confirm that PER inhibited pAkt activity in HNSCCs.

### 3.2. Effect of Perifosine on Cells Growth and Survival

**Figure 2:**
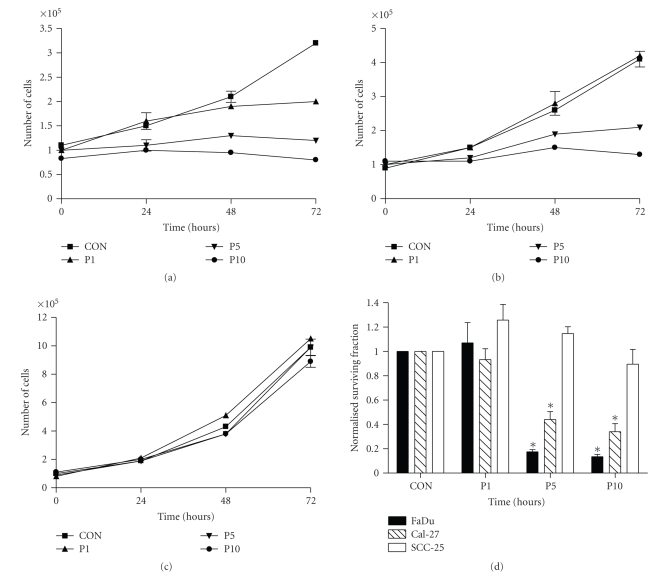
Effect of perifosine (PER) in head and neck cancer cell growth and survival. FaDu (a), Cal-27 (b), and SCC-25 (c) cells were treated with 1, 5, and 10 *μ*mol/L PER and grown for 72 hours. (d) Cells were treated with 1–10 *μ*M PER for 24 hours then plated for clonogenic survival. Clonogenic cell survival data were normalized to control (CON). Error bars represent ± 1SD of *N* = 4–6 experiments performed on different days with at least 2 cloning dishes taken from 1 treatment dish. ∗, *P* < .05 versus control.

To investigate the effect of PER on human head and neck squamous carcinoma cell growth, FaDu, Cal-27, and SCC-25 cells were treated with increasing doses of PER (1–10 *μ*M) and then counted at 0, 24, 48, and 72 hours after treatment. PER at 1, 5, and 10 *μ*M inhibited FaDu cell growth after 72 hours (Figure 2(a)) while only 5 and 10 *μ*M PER inhibited Cal-27 growth after 72 hours (Figure 2(b)). SCC-25 cells did not respond to any of the PER doses over a 72-hour period (Figure 2(c)). When we analyzed clonogenic survival after 24 hours of treatment with PER, we observed that 1 *μ*M PER did not affect survival in any of the cells lines but 5 and 10 *μ*M PER significantly decreased survival in FaDu and Cal-27 cells compared to control (*P* < .01, Figure 2(d)). SCC-25 cells were again resistant to PER treatment with 10 *μ*M PER causing a slight but nonsignificant decrease in SCC-25 cells compared to control (Figure 2(d)). These results show that PER causes growth arrest and cytotoxicity in HNSCC cells but these affects are dose dependent and vary by cell type.

### 3.3. Perifosine Induced Disruptions in Glutathione Metabolism Consistent with Oxidative Stress

**Figure 3:**
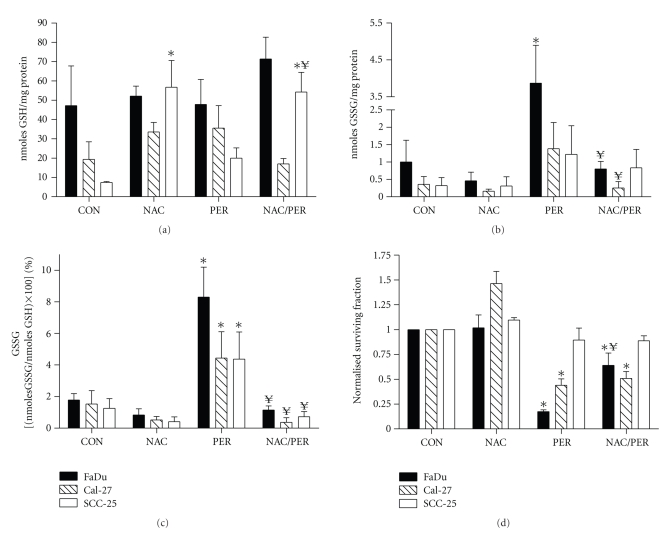
Effect of perifosine (PER) and N-acetyl-cysteine (NAC) on total glutathione (GSH) levels (a), glutathione disulfide (GSSG) levels (b), percentage glutathione disulfide (%GSSG) levels (c), and cytotoxicity (d) in head and neck cancer cells. Cells were treated with 5 *μ*M PER (FaDu and Cal-27) or 10 *μ*M PER (SCC-25) for 24 hours with or without treatment with 20 mM NAC for 1 hour before and during PER exposure. (a)–(c) Cells were harvested for glutathione analysis using the spectrophotometric recycling assay. Error bars represent ± 1SD of *N* = 4 experiments. (d) Cells were analyzed for clonogenic survival and the data were normalized to control (CON). Error bars represent ± 1SD of *N* = 3 experiments performed on different days with at least 2 cloning dishes taken from 1 treatment dish. ∗, *P* < .001 versus control; ¥, *P* < .001 versus respective treatment without NAC.

We examined if oxidative stress could be contributing to the growth inhibitory and cytotoxic effects of PER by measuring glutathione (GSH/GSSG) levels in the cells. The GSH/GSSG redox couple represents a major small molecular weight thiol-disulfide redox buffer in cells [27]. The amount of total GSH that was oxidized (GSSG) was used to calculate percentage GSSG (%GSSG). Consequently, an increase in %GSSG is believed to signify a shift towards a more highly oxidizing intracellular environment indicative of oxidative stress [27]. We analyzed GSH/GSSG levels in FaDu, Cal-27, and SCC-25 cells after treatment with 5 *μ*M PER for 24 hours. We chose to further analyze the effects of 5 *μ*M PER in FaDu and Cal-27 cells because it was a clinically achievable dose and well under mean steady state plasma concentrations (16.2 *μ*M) achieved in patients with solid tumors after a maximum tolerated dose of 200 mg PER per day [12]. We analyzed the effects of 10 *μ*M PER in SCC-25 cells because this was the only dose that showed slight cytotoxicity in the clonogenic cell survival assay (Figure 2(d)). PER caused an increase in total GSH levels in Cal-27 and SCC-25 cells compared to control which suggests that PER induced the synthesis and accumulation of total GSH in an attempt to maintain the redox buffering capacity of the intracellular reducing environment (Figure 3(a)). PER did not significantly change total GSH levels in FaDu cells (Figure 3(a)). Increases in GSSG were observed when all cell lines were treated with PER although this effect was only significant in FaDu cells (Figure 3(b)). When we calculated the effect of PER on %GSSG, all 3 cell lines demonstrated significant increases in %GSSG compared to control cells with FaDu showing the greatest increase in %GSSG and SCC-25 showing the least (Figure 3(c)). These results support the hypothesis that the toxicity of PER may be in part mediated by disruptions in GSH and/or thiol metabolism consistent with causing oxidative stress.

### 3.4. PER-Induced Disruptions in Glutathione Metabolism and Cytotoxicity Are Inhibited by NAC

To further analyze the involvement of oxidative stress in PER-induced cytotoxicity, FaDu, Cal-27, and SCC-25 cells were treated with 20 mM of the thiol antioxidant N-acetyl cysteine (NAC) for 1 hour before and during exposure to PER, then analyzed for GSH/GSSG levels and clonogenic survival. Treatment with NAC alone significantly increased GSH levels in SCC-25 cells and also increased GSH in the presence of PER in SCC-25 cells (Figure 3(a)). Additionally, NAC suppressed the PER-induced increase in GSSG in all 3 cell lines with FaDu and Cal-27 reaching significance (Figure 3(b)). However, PER-induced increases in %GSSG were significantly suppressed by NAC in all 3 cell lines which were comparable to NAC alone (Figure 3(c)). When we analyzed the effect of NAC on PER-induced cytotoxicity, NAC partially but significantly rescued the cytotoxicity induced by the combination of PER (36%: NAC + PER versus 83%: PER, *P* < .05) in FaDu cells (Figure 3(d)). NAC did not significantly rescue Cal-27 and SCC-25 cells from PER-induced cytotoxicity (Figure 3(d)). Taken together, Figure 3 supports the hypothesis that PER induces disruptions in thiol metabolism consistent with oxidative stress, which was reversed by NAC, and PER-induced cytotoxicity in FaDu cells may be due in part to increases in oxidative stress.

### 3.5. PER-Induced Cytotoxicity Is Enhanced by Buthionine Sulfoximine

**Figure 4:**
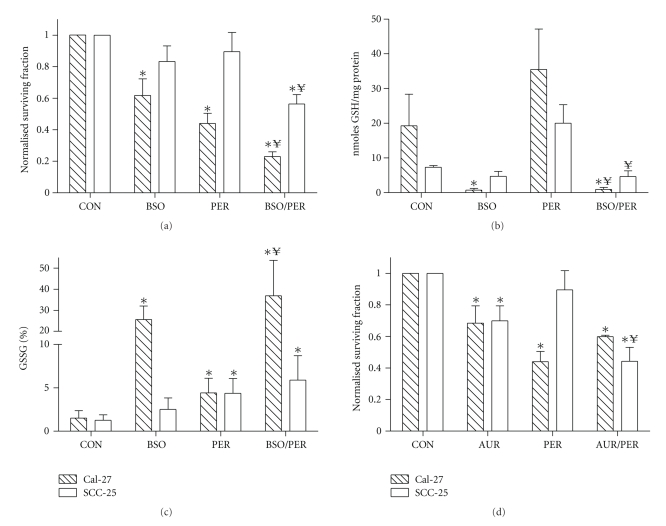
Effect of inhibitors of glutathione and thioredoxin metabolism on perifosine (PER) toxicity in head and neck cancer cells. (a) Cal-27 and SCC-25 cells were treated with 5 *μ*M PER (Cal-27) or 10 *μ*M PER (SCC-25) for 24 hours with or without treatment with 1 mM buthionine sulfoximine (BSO) for 1 hour before and during PER exposure. (b)-(c) Cells were treated as stated above and harvested for total glutathione (GSH) levels (b), and percentage glutathione disulfide (%GSSG) levels (c) using the spectrophotometric recycling assay. Error bars represent ± 1SD of *N* = 3 experiments. (d) Cal-27 and SCC-25 cells were treated with 5 *μ*M PER (Cal-27) or 10 *μ*M PER (SCC-25) for 24 hour with or without treatment with 0.5 *μ*M Auranofin (AUR) for 1 hour before and during PER exposure. Clonogenic cell survival data were normalized to control (CON). Error bars represent ± 1SD of *N* = 3 experiments performed on different days with at least 4 cloning dishes taken from 1 treatment dish. ∗, *P* < .001 versus control; ¥, *P* < .05 versus respective treatment without BSO or AUR.

To determine if GSH depletion would further enhance the cytotoxicity induced by PER, Cal-27, and SCC-25 cells were treated with 1 mM BSO, which is an inhibitor of GSH synthesis, for 1 hour before and during treatment with PER for 24 hours then analyzed for clonogenic survival and GSH/GSSG. We did not study the effect of BSO on PER-induced cytotoxicity in FaDu cells since PER caused such extensive cell killing as a single agent in this cell line. The results indicate that the cell killing observed with PER in Cal-27 was significantly enhanced by BSO (77%: BSO + PER versus 56%: PER, *P* < .05, Figure 4(a)). Additionally, SCC-25 cells which were initially resistant to PER treatment (Figure 2(d)) became sensitized to PER in the presence of BSO (44%: BSO + PER versus 11%: PER, *P* < .05, Figure 4(a)), but this sensitization was not comparable to the cytotoxicity induced by PER in FaDu (83%) and Cal-27 cells (56%, Figure 2(d)). BSO alone and in the presence of PER significantly depleted total GSH to less than 10% of control cells in Cal-27 but only depleted 35% of total GSH in SCC-25 cells (Figure 4(b)). Furthermore, BSO alone and in combination with PER significantly increased %GSSG in Cal-27 cells compared to control cells which was not observed in SCC-25 cells (Figure 4(c)). These data show that significant depletion of glutathione (GSH) with BSO enhances the cytotoxicity observed with PER and the extent of sensitization may be due to the degree of GSH depletion and induction of GSSG.

### 3.6. PER-Induced Cytotoxicity Is Enhanced by Auranofin

Since BSO did not significantly enhance PER-induced cell killing in SCC-25 cells to that of PER-induced cell killing observed in FaDu and Cal-27 (Figure 2(d)), we determined if inhibition of thioredoxin (Trx) metabolism would enhance PER-induced cell killing in SCC-25 cells and compared the results to those of Cal-27. We pretreated cells with 0.5 *μ*M AUR for 1 hour before and during exposure to PER (Figure 4). We chose this dose of 0.5 *μ*M AUR because it was shown to inhibit thioredoxin reductase (TrxRed) activity by approximately 50% in Cal-27 and SCC-25 cells compared to control cells (Table 1). AUR caused about 30% cell killing in both cell lines compared to control (Figure 4). Interestingly, AUR did not further enhance PER-induced cell killing in Cal-27 cells but AUR did sensitize SCC-25 cells to PER-induced cell killing which was now comparable to PER-induced cell killing in Cal-27 cells (56%: AUR + PER [SCC-25] versus 56%: PER [Cal-27], Figure 4). Table 1 shows that AUR alone and in combination with PER did in fact inhibit TrxRed activity in Cal-27 and SCC-25 cells. PER alone did not affect TrxRed activity in any of the cell lines (Table 1). The data in Figure 4 and Table 1 suggest that inhibition of GSH and Trx metabolism pathways enhance sensitivity to PER.

### 3.7. Thiol Antioxidant Status Predicts Response to Perifosine

**Figure 5:**
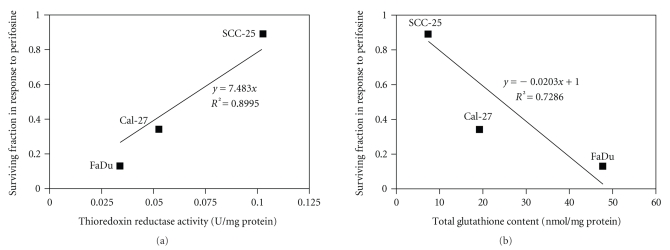
Association of perifosine-(PER-) induced cytotoxicity with glutathione (GSH) content and thioredoxin reductase activity (TrxRed) in head and neck cancer cells. FaDu, Cal-27, and SCC-25 cells were analyzed for mean baseline TrxRed activity (a) and mean total GSH content (b) and compared to their respective mean surviving fraction in response to 5 *μ*M PER (FaDu and Cal-27) or 10 *μ*M PER (SCC-25).

Since each head and neck cell line evaluated in these studies varied in their response to PER (Figures 1  and 2), and we demonstrated that disrupting GSH or Trx antioxidant pathways sensitized certain cell lines to PER (Figure 4), we determined if GSH or TrxRed activity predicted response to PER. We plotted the surviving fraction of each cell line in response to PER as a function of TrxRed activity at control levels (Figure 5(a)) and total GSH content at control levels (Figure 5(b)). The results showed that TrxRed activity significantly correlated with PER-induced cytotoxicity, with FaDu, which demonstrated the greatest cell killing in response to PER (Figure 2(d)), having the least TrxRed activity (Figure 5(a)), and SCC-25 cells which were resistant to PER (Figure 2(d)), having the highest level of TrxRed activity (*r* ^2^ = 0.8995, Figure 5(a)). Additionally, total GSH levels also correlated with PER-induced cytotoxicity, with FaDu cells having high GSH levels compared to the other cell lines and SCC-25 cells having low GSH levels (*r* ^2^ = 0.7286, Figure 5(b)). These data suggest that cells with high TrxRed activity and low total GSH levels may be resistant to PER and cells with low TrxRed activity and high GSH content may be sensitive to PER.

## 4. Discussion

PER as a single agent has shown favorable responses in patients with advanced soft tissue sarcomas [14] and Waldenstrom macroglobulinemia [28]. However, responses to PER in patients with common solid tumors have been disappointing and have not justified the further investigation of PER as a single agent. In this study we investigate the role oxidative stress plays in the mechanism of PER and show that sensitivity to PER may be enhanced by disrupting thiol antioxidant metabolism pathways and increasing oxidative stress in head and neck cancer cells.

Akt expression and hyperactivation is a frequent event in HNSCC and strongly correlates with disease progression [8, 29]. The HNSCC cell lines used in this study, FaDu, Cal-27, and SCC-25 all expressed the activated form of Akt (pAkt, Figure 1), which was inhibited by 24 hours treatment with 5 *μ*M PER. Although pAkt was inhibited in all cases, sensitivity to PER varied among the cell lines with FaDu exhibiting the greatest growth inhibition and cytotoxicity and SCC-25 exhibiting no response (Figure 2(d)). Nevertheless, these data support findings by Kondapaka et al. (2003), who showed that 5 *μ*M PER for 24 hours inhibited pAkt expression and growth in PC-3 prostate carcinoma cells [21] and Patel et al. who showed antiproliferative effects with 0.6–8.9 *μ*M PER in head and neck cancer cells [30]. Additionally we observed that pAkt was expressed at different levels in the 3 cell lines with FaDu showing the highest expression and SCC-25 showing the least (Figure 1). Since FaDu cells with the highest expression of pAkt were the most sensitive to the Akt inhibitor PER and SCC-25 cells with relatively low pAkt expression were resistant to PER, it is possible that tumors with high pAkt expression are more susceptible to Akt pathway inhibitors than tumors with low pAkt expression. 

Activation of the Akt pathway is crucial for cell survival and cellular redox status is involved in the reversible activation and inactivation of this pathway [4, 31, 32]. For example, moderate levels of ROS activate Akt pathway signaling and promote cell survival, but high or chronic oxidative stress inhibits this pathway resulting in apoptosis [4, 31–34]. Since cancer cells are under increased metabolic oxidative stress compared to normal cells and the Akt pathway may be activated for survival under these oxidizing conditions, we proposed that inhibition of the Akt pathway with PER would increase oxidative stress to such an extent that would render cancer cells sensitive to further increases in oxidative stress. 

We investigated the effects of PER on oxidative stress by analyzing glutathione (GSH/GSSG) levels. The glutathione system is a major intracellular redox buffer in the cell and is involved in the detoxification of H_2_O_2_ and organic hydroperoxides [27] and the ratio of GSH to GSSG can be used as a reflection of intracellular redox status [27]. PER induced significant increases in %GSSG in all 3 cell lines compared to control cells (Figure 3(c)) which indicated an increase in oxidative stress and suggests that inhibition of Akt may be involved in increasing oxidative stress. To further support this idea, the thiol antioxidant NAC was able to completely suppress the increase in %GSSG in all 3 cell lines (Figure 3(c)). Additionally, NAC was able to partially but significantly reverse the cytotoxicity induced by PER in FaDu cells suggesting that increased oxidative stress was involved in PER-induced cytotoxicity in this cell line (Figure 3(d)).

To further probe the role of GSH in the effects of PER, we used BSO, an inhibitor of glutamate cysteine ligase, which is believed to be the rate-limiting enzyme in the synthesis of GSH [35, 36] in Cal-27 and SCC-25 cells. Previous studies in our laboratory have shown that BSO significantly depleted GSH pools in breast and head and neck cancer cells while sensitizing cancer cells to chemotherapy agents [37, 38]. BSO has also been used in clinical trials for cancer therapy to enhance the cytotoxicity of chemotherapeutic agents [39]. In the present studies, BSO was found to significantly increase the cytotoxicity induced by PER in Cal-27 cells (Figure 4(a)). As expected, BSO significantly decreased total GSH levels and increased %GSSG in Cal-27 cells as a single agent and in combination with PER (Figure 4(b)), which suggests that inhibition of GSH synthesis further enhanced the oxidative stress induced by PER and further sensitized these cells to the toxicity of PER in Cal-27 cells. 

Overall SCC-25 cells were more resistant to PER treatment than FaDu and Cal-27 cells (Figures 1  and 2). We also observed that BSO was not as effective in SCC-25 cells as in Cal-27 cells at sensitization to PER (Figure 4(a)), which was evident in the lack of significant GSH depletion in this cell line (Figure 4(b)). Although there was a trend toward an increase in %GSSG in BSO + PER compared to PER alone in SCC-25 cells (Figure 4(c)), this increase was not significant and was not nearly as great as the BSO + PER-induced increase in %GSSG observed in Cal-27 cells (Figure 4(c)). These observations suggest that PER-induced cytotoxicity in SCC-25 cells is less dependent on the glutathione/glutathione peroxidase system than in Cal-27 cells. Preliminary experiments in our laboratory support these observations and show that PER induced an increase in GPx activity in Cal-27 cells but not SCC-25 cells (data not shown). Therefore, we propose that other antioxidant systems, such as the thioredoxin (Trx) system may be involved in PER-induced cytotoxicity in SCC-25 cells. 

The Trx system is a highly conserved ubiquitous system comprised of thioredoxin reductase (TrxRed), thioredoxin (Trx), thioredoxin peroxidases (a.k.a., peroxiredoxins), and NADPH [40]. The Trx system plays an important role in the redox regulation of multiple intracellular processes and resistance to cytotoxic agents that induce oxidative stress [41, 42]. TrxRed is a selenocysteine-containing protein that catalyzes the reduction of Trx using NADPH as a reducing agent [40]. TrxRed has been shown to initiate signaling pathways in response to oxidative stress that play a role in protecting the cell from oxidative stress and is therefore believed to be a potential target for cytotoxic agents that induce oxidative stress [40, 43, 44]. 

To investigate the role of Trx metabolism in SCC-25 cells we used Auranofin (S-triethylphosphinegold(I)-2,3,4,6-tetra-O-acetyl-1-thio-b-Dglucopyranoside (AUR)), which is a relatively specific inhibitor of TrxRed (Table 1). AUR belongs to the gold(I)-based drug class utilized in the treatment of rheumatoid arthritis [45] and has been shown to stimulate the mitochondrial production of hydrogen peroxide [46, 47]. AUR significantly sensitized SCC-25 cells to PER (Figure 4(d)) to a greater extent than BSO (Figure 4(a)), which was comparable to the PER-induced cytotoxicity seen in Cal-27 (Figure 2(d)). Interestingly, AUR did not significantly sensitize Cal-27 cells to PER (Figure 4(d)). This further supports the idea that the Trx pathway was more involved in PER-induced cytotxicity in SCC-25 cells than Cal-27 cells, in which the GSH pathway appeared to be more important. 

We expected that BSO or AUR would sensitize Cal-27 and SCC-25 cells to PER to a greater extent than what we observed in Figures 4(a) and 4(d)  based on the fact that both BSO, AUR and PER all induce oxidative stress as single agents and should be mechanistically linked. However, we do acknowledge that more extensive analysis of dose responses for BSO + PER and AUR + PER is needed (i.e., isobologram analysis) and our data shown in Figure 4 does not address this issue of mechanistic linkage. On the other hand, our data strongly suggests the opposite, in that BSO or AUR in combination with PER may be mechanistically unlinked since BSO + PER (in Cal-27 and SCC-25) and AUR + PER (in SCC-25) appear to be additive (Figures 4(a)  and 4(d)). BSO inhibits the synthesis of GSH, AUR inhibits TrxRed activity and PER inhibits Akt pathway signaling, therefore, the mechanism of action of these agents is not linked. However, BSO, AUR and PER all have significant effects on oxidative stress which further justifies the need for more extensive dose response analysis of these drugs to determine additivity or synergy.

It is important to note that SCC-25 cells possess twice as much TrxRed activity as Cal-27 cells and that Cal-27 cells possess about twice the amount of total GSH compared to SCC-25 cells (Table 1, Figure 4). This led us to propose that total GSH and TrxRed activity may predict response to PER in head and neck cancer cells. To gain some insight into this relationship we plotted TR activity and total GSH of each cell line at control levels against the surviving fraction in response to PER (Figure 5). The results suggested that high TrxRed activity and low total GSH (as in SCC-25 cells) rendered the cells resistant to PER treatment, while low TrxRed activity and high total GSH (as in FaDu cells) rendered cells sensitive to PER (Figure 5). These results support data by Ceccarelli et al. [48], who showed that lung cancer cells with high Trx expression levels had a more aggressive phenotype, but were more sensitive to Trx inhibition than cells with low Trx expression levels. These results also may account for why TrxRed is upregulated in many tumor cells and supports the speculation that drug-resistant cells may be more susceptible to the inhibition of TrxRed to promote cytotoxicity and susceptibility to other chemotherapeutic drugs. Additionally, our preliminary experiments have shown higher GPx activity in FaDu cells compared to Cal-27 and SCC-25, and GPx activity appeared to correlate with sensitivity to PER similar to that shown in Figure 5(b) (data not shown). We are currently repeating these experiments, but these encouraging results further suggest that there may be a higher level of GSH metabolism in FaDu cells compared to SCC-25 cells.

 Overall, the data provided here support the conclusion that PER induces oxidative stress in HNSCC cells and disrupting thiol antioxidant metabolic pathways enhances susceptibility to PER via oxidative stress. These data also support the speculation that TrxRed activity and total GSH levels may help to predict response to PER. Finally, these data provide a biochemical rationale for the use of inhibitors of GSH and Trx metabolism in combination with PER to enhance susceptibility of HNSCC cells to clonogenic cell killing by PER in combined modality cancer therapies.

## Figures and Tables

**Table 1:** 

	Thioredoxin reductase activity (U/mg protein)			
Cell line	CON	AUR	PER	AUR/PER
Cal-27	0.05 ± 0.02	0.03 ± 0.01	0.07 ± 0.01	0.01 ± 0.001
SCC-25	0.10 ± 0.003	0.05 ± 0.04	0.09 ± 0.02	0.04 ± 0.04

## Abbreviations

PER: Perifosine
AUR: Auranofin
BSO: L-buthionine-[S,R]-sulfoximine
NAC: N-acetyl cysteine
GSH: Glutathione
GSSG: Glutathione disulfide
TrxRed: Thioredoxin reductase
Trx: Thioredoxin.
